# Graduates from a reformed undergraduate medical curriculum based on *Tomorrow's Doctors *evaluate the effectiveness of their curriculum 6 years after graduation through interviews

**DOI:** 10.1186/1472-6920-10-65

**Published:** 2010-09-29

**Authors:** Simon D Watmough, Helen O'Sullivan, David CM Taylor

**Affiliations:** 1 Centre for Excellence in Developing Professionalism, School of Medical Education University of Liverpool, Cedar House, Ashton Street, Liverpool, L69 3GE. UK

## Abstract

**Background:**

In 1996 Liverpool reformed its medical curriculum from a traditional lecture based course to a curriculum based on the recommendations in Tomorrow's Doctors. A project has been underway since 2000 to evaluate this change. This paper focuses on the views of graduates from that reformed curriculum 6 years after they had graduated.

**Methods:**

Between 2007 and 2009 45 interviews took place with doctors from the first two cohorts to graduate from the reformed curriculum.

**Results:**

The interviewees felt like they had been clinically well prepared to work as doctors and in particular had graduated with good clinical and communication skills and had a good knowledge of what the role of doctor entailed. They also felt they had good self directed learning and research skills. They did feel their basic science knowledge level was weaker than traditional graduates and perceived they had to work harder to pass postgraduate exams. Whilst many had enjoyed the curriculum and in particular the clinical skills resource centre and the clinical exposure of the final year including the "shadowing" and A & E attachment they would have liked more "structure" alongside the PBL when learning the basic sciences.

**Conclusion:**

According to the graduates themselves many of the aims of curriculum reform have been met by the reformed curriculum and they were well prepared clinically to work as doctors. However, further reforms may be needed to give confidence to science knowledge acquisition.

## Background

There has been widespread reform around the world in undergraduate medical curricula in recent years [1]. In the United Kingdom (UK) medical schools have introduced radical changes to the content of curricula due to recommendations contained in *Tomorrow's Doctors *[2-4] by the General Medical Council (GMC) which reflected world wide trends in medical education. The GMC called for an end to factual overload with integration of basic and clinical sciences and a move away from didactic teaching to encourage problem solving, critical thinking and life-long learning. Due to the changing expectations of patients the GMC also recommended the introduction of communication skills tuition and to reflect modern health care an increase in community based medical education. Despite this, and the fact curriculum reform has continued at pace around the world [5] there have been few studies evaluating the long term impact of these recommendations. UK studies have been carried out examining the competencies of graduates of reformed curricula in first postgraduate year [6], and some quantitative studies have looked at the impact of curriculum reform in other countries [7-9] but few qualitative studies have been undertaken evaluating the longer term impact of curriculum reform. This paper summarises 45 interviews which took place with 45 graduates from the first two cohorts to graduate from the University of Liverpool's reformed medical curriculum. The aim of the interviews was to ask graduates from a reformed curriculum to evaluate the effectiveness of their undergraduate education.

### The Curriculum Evaluation Project

The interviews stem from a project called "The Liverpool Medical Curriculum Evaluation Project". In 1996 Liverpool reformed its medical programme from a traditional lecture-based programme to a curriculum based on the recommendations in *Tomorrow's Doctors *[2]. The project initially involved evaluating the impact of curriculum reform by gathering views on the content of the reformed medical curriculum and examining the perceived competencies of the final 2 cohorts of the traditional curriculum and the first two cohorts of the reformed medical curriculum working as first year graduates [10-12]. This study aims to build on the previous work undertaken and ascertain how effective graduates who studied on a curriculum based entirely on *Tomorrow's Doctors *perceived their undergraduate medical education was 6 years after graduation. Working for 6 years allowed close enough recall of their undergraduate programme but gave them enough postgraduate experience of making clinical decisions as doctors past the closely supervised first postgraduate year to make a significant evaluation of the effect of their undergraduate education.

### The Liverpool Curriculum

Prior to 1996 the University of Liverpool had a very traditional medical curriculum which was lectured-based with distinct clinical and pre clinical parts and little integration between the different sections [13]. From 1996 problem-based learning (PBL) replaced lectures as the main learning activity in years 1-4. During the PBL tutorials students develop their own learning objectives that they research independently supported by plenary sessions, on line resources and a Human Anatomy Resource Centre (HARC). Science teaching is integrated throughout the course with clinical exposure increasing year by year. Students learn practical and clinical skills in the University's Clinical Skills Resource Centre from the first semester and in hospital clinical skills centres in later years of the course. Approximately 30% of undergraduate clinical attachments take place in the community compared with three weeks in the traditional medical curriculum and Student Selected Components (SSCs) account for about 25% of the course. Students undertake timetabled communications classes in years 1 and 2 and are assessed on communication and clinical skills throughout the course. Final exams take place at the end of 4^th ^year and the final year is an apprentice year designed to prepare students for practice including an 8 week "shadowing" placement, an A & E attachment, a GP placement and 2 selectives in advanced medical practice (SAMPs) which they chose themselves with students undertaking assessment via a portfolio in the final year.

## Methods

Ethical approval was gained from the National Health Service (NHS) COREC Liverpool Research Ethics Committee to contact the graduates for their consent to take part in this project. All doctors in the UK have a statutory duty to be registered with the General Medical Council (GMC). In January 2007 and spring 2008 the GMC was contacted to supply registration numbers and contact addresses for students who graduated in 2001 and 2001 respectively (the first two cohorts to graduate from the reformed medical curriculum). 330 graduates were contacted, although details of 30 were unavailable, possibly because they had changed name (maybe due to marriage) or were no longer registered with the GMC.

During 2007 and 2008 the graduates were sent a letter and consent form inviting them to take part in both the questionnaire and interview parts of the project. 71 graduates volunteered to take part in an interview. They were contacted three times via email from SW. If it proved impossible to arrange a mutually convenient time they were not contacted again. A total of 45 interviews, typically lasting 30-40 minutes took place. 34 of the interviews took place face to face either in the hospital, surgery, home of the interviewee or office of SW and 11 interviews for those doctors who lived outside the Liverpool area took place via telephone. 25 interviewees were 1^st ^cohort graduates and 20 were 2^nd ^cohort. 18 interviewees were GPs, 15 were physicians, 7 were surgeons, 3 were psychiatrists and 2 were anaesthetists. 4 were taking time of their training undertaking research projects at the time of the interview. The 2001 cohort interviews took place between March and August 2007 and the 2002 cohort interviews took place between October 2008 and March 2009 so all graduates had been working for approximately 6 years.

### Analysis

The questions were based on the questions used with the focus groups which were held with these doctors as first year postgraduates and consultants who supervised them, during the early stages of the evaluation project [14,15] and the expectations of competencies of doctors according to the GMC [2-4]. These were then were put into a series of broad questions (see below) encompassing their preparedness to undertake skills which are generic across all specialties such as communicating effectively and taking a history and examination. Also, questions were asked, specifically about the content of their undergraduate course. All interviewees had already returned questionnaires [16] prior to these interviews taking place and from the responses to the questionnaires it was clear that these doctors were able to assess their undergraduate programme.

The interviewer was a non-clinician researcher (SW), who, prior to the interviews, was generally unknown to the interviewees. This, together with him not being in a management position within the University or the National Health Service (NHS) reduced the possibility of bias [17] during the interviews. The interviews were tape-recorded and then transcribed *verbatim *by SW. The analysis was based on the framework approach which allows the objectives of the research to be determined prior to data collection. These prior objectives were covered in the basic questions to all interviewees which included: what was your science knowledge like; how did you learn your communication skills; how well prepared were you to work as a junior doctor; were you well trained in history and examination skills; did you graduate with the necessary research skills; did you receive adequate training undertaking practical procedures on patients; what do you think about the structure/content of the course; what did you feel about the amount of General Practice in your course; what were the strengths and weaknesses of your course; is there anything else you would like to add about your time as an undergraduate?

The framework approach involves clear stages of data analysis which were applied to the analysis of these interviews: familiarisation; identifying a thematic framework; indexing; charting; mapping and interpretation. The tapes and transcripts were re-listened to and re read for familiarisation. The thematic framework was then identified by examining the priori issues (in this case the questions) and issues raised by the interviewees. The data was then clearly coded and the text was indexed by using descriptors alongside various passages in the transcriptions. The data was then charted alongside the appropriate part of the thematic framework and finally, the charts were mapped to explore associations between the themes and examine the original research objectives and emerging themes. The transcripts were originally analysed by SW using steps identified above [18]. The other authors of this paper independently read through the transcriptions to reduce biases and validate the findings. There were no major differences in the views of the co-authors with those of SW of the themes and emerging issues. Using the framework approach meant that all the themes we considered important were covered in the interviews which helped gain saturation of themes. Kvale [19] has stated that 15 - 20 interviews with a group of people with similar backgrounds is often enough to gain saturation of themes so 45 interviews ensured no important views were missed out. The questions, "what were the strengths and weaknesses of your course?" and "is there anything else you would like to add?" ensured interviewees could raise issues which were important to them which may not have been covered previously.

## Results

### Knowledge base

There were mixed views on their knowledge base and some were very much specialty dependent - for example a microbiologist believed there should have been more microbiology and biochemistry and some surgeons wanted more anatomy teaching.

A small number felt there no problems at all:

"I am aware of the controversies but I think my knowledge base is absolutely fine"

The majority, though felt their knowledge was weak.

"I felt I lacked confidence - my knowledge was based on diseases not anatomy and physiology."

"it wasn't at all good. There was no anatomy. It was disgraceful."

"I felt it was pretty poor, really. The sciences were just never emphasized at medical school."

Although the majority were glad they didn't have to undergo the amount of science lectures in the traditional medical course they would have been happier if they had received more tuition in the sciences.

"I felt like "A" (*school*) level biology and chemistry didn't get built on, it was good that we didn't learn the Krebs cycle and all that rubbish but it didn't take you on to another level."

They felt they had enough knowledge to work as first year postgraduates but had to study harder when it came to taking professional postgraduate exams compared with traditionally educated peers.

".. it does furnish you to be a qualified doctor, but I always said we would struggle when we got postgraduate exams and I don't know if this became a self fulfilling prophecy, but part one of the exam I did struggle with - it was fine when I got through that and onto the clinical paper but I did find I was lacking basic sciences."

They had all been told by their consultants on clinical placements that PBL graduates are lacking in science knowledge.

"as a group we felt we didn't have enough particularly in anatomy....but you found out you didn't need it...the main problems were the consultants asking questions on ward rounds.."

They attributed their perceived lack of science knowledge to learning sciences within a PBL system which they saw as too "unstructured."

"our knowledge wasn't good because you can't really expect 18/19 year olds just to get on with it....there was no real theoretical background."

"I would say we were short in that area... the problem with teaching yourself is you are not sure what to teach yourself."

There were some contradictions - many did feel that they had good knowledge of one or two of the sciences, for example physiology but then felt weak on others. Physiology was seen as being their strongest science with anatomy seen as the weakest followed by pharmacology. The majority of the interviewees, though, said that they gained an understanding of disease processes as undergraduates. Only a small number didn't gain this understanding until working as postgraduates.

"the case scenario made it more real and translates what you learned on paper....the anatomy would remind you of the physiology...it was more linked."

"understanding disease processes was fine and we were clinically competent with a good knowledge of clinical medicine but not the individual sciences."

### Communication skills

The majority of interviewees felt that they were good communicators and they attributed this to the communication skills tuition in the course.

"I do think you have to learn to be a good communicator, I think everyone has a basic level of communication and some are better than others when we start... I know we all hated the role play but it is something we still do now.."

Although a large number of interviewees did feel that it was perhaps too many of these classes or some of them were a bit "over the top" or that they didn't enjoy them. However, even then they did have their uses.

"My perception is they are a safety net for those people who don't have the skills.."

But even those who admitted that it was good to have them to make sure that all doctors at least had the basics in what the graduates recognised was a very important part of being a doctor.

"I found the communication sessions absolutely excruciating because I didn't find it easy but I think without them I would have found the transition very hard so they were good for me although I didn't enjoy them at all."

### History and examination

There was near unanimity amongst the doctors that they had received good preparation in this area from their undergraduate programme and it was a strength of the curriculum.

"I think it is one of the strongest points and we could take a good history and examination."

"The clinical teaching in the university was a good base and on wards rounds and stuff with the examinations we did."

There were two clear parts of the programme where they felt they had learned these skills. The first was in the clinical skills resource centre and then they said they had the chance to practice these skills in the latter clinical placements in the course and many had said this came from some "traditional bedside teaching" from consultants. It was from this 4^th ^and 5^th ^year teaching in particular that they managed to learn about differential diagnoses.

"The 4^th ^year was really good for that, by the end of the 4^th ^year we had picked up differential diagnoses from our consultants."

"I learned proper history taking in clinical skills.... everything was structured...we learned the differential diagnoses and management later in the course.."

The graduates really enjoyed the Clinical Skills Resource Centre - not only did they find the teaching fun and relevant they also found it re assuring to have some formal teaching whilst working through the "uncertain" PBL. It was also useful for learning practical skills.

"I think the skills lab was definitely very useful and we did everything from cannulation to examination and all these kinds of things... I think that definitely gives you the confidence to go out and do it."

"it was a joy to have a session where you knew that you were going to learn and there was some kind of syllabus and that you were going to get something out of the sessions."

### Research skills

It was also discussed with all the interviewees whether they felt they had learned good research and "self directed" skills which is a key aim of introducing PBL into a medical curriculum. From the PBL and SSCs all the interviewees felt that they had these skills, particularly in literature searching and many of the interviewees said they had undertaken audits or were aware of them as medical students. They said they were comfortable to go away and look things up that they didn't know. These skills were also useful for revising for postgraduate exams and were useful skills to have for all doctors:

"...it makes you aware that research is there to be done and gives you confidence to do it, to talk about it and then go away and do it."

"I think in terms of literature searches and looking things up - yes...you do pick up about research methods...useful to have those skills even if you don't use them every day.. it does encourage self directed learning and it is useful to have those skills."

However, this wasn't always necessarily seen as totally positive and some said whilst they appreciated these skills they still wanted more "structure".

"It was very difficult to start with because you come out of school and everything was handed to you on a plate..... and I found it really difficult at first doing self directed learning but I found it suited my style of learning so liked it."

### General Practice

The general view was that it was beneficial that community teaching had increased compared with the traditional medical course and even those who felt it had been increased maybe too much could appreciate the benefit of community attachments. Only a small number of those interviewed felt there was now too much community teaching.

"Personally I think it was rammed down our throats a bit much at the time.."

GP was seen as useful for practising clinical skills such as communicating and examination and all the graduates wherever they worked at the time of interview felt they understood the relationship between primary and secondary care.

"Knowing the stresses the GPs are under when you are in MAU (Medical Assessment Unit) or whatever....you can see what the GP might be thinking with the pressures on their time and yeah it really defined the roles of primary and secondary care and was very useful.."

"The GP was neglected in the past..... not a bad thing it increased....we certainly understood the relationship between GP and the hospitals.... but I found it boring compared with surgery!"

All the General Practitioners in the study said they felt very well prepared for postgraduate training.

### Practical skills/Prepared for the role of junior doctor

All the interviewees felt they have received good preparation for working as junior doctors. In fact it was striking how little anxiety there was about this.

"We learned all the skills we needed so we weren't scared when we started, we did all the venflons and gasses, saw how the team worked and what to do.."

"I don't remember there being a big jump at all, we turned up and got on with the job."

They also felt they had been well prepared to undertake the basic practical procedures on patients that are an important part of the role of a junior doctor.

The doctors indicated a number of factors why they felt they had been so well prepared to work as junior doctors. The most important reason was the 8 week "shadowing" attachment in the final year.

"the shadowing made it a lot easier because I didn't feel I was starting a job, it felt like I was doing the job when I started.... I was in the same hospital, same ward same consultant..... my first time was 9 pm at night but I felt comfortable and fitted right in."

Other attachments were in the final year were also seen as important and in particular the A & E attachment was seen as very useful. The final year not only gave the opportunity to practice and learn what the job entailed and some of the more mundane aspects of the role but also gave the opportunity to practice communication, history and examination skills. The fact they had received good grounding in these skills earlier in the course in the Clinical Skills Resource Centre helped them practise in the final year.

"yeah the cannulation, catheterisation we had lots of practice on dummies then we were supervised doing these things by junior doctors when we shadowed."

Having exams at the end of the 4^th ^year was seen as extremely useful in preparing them to work as junior doctors because they could get on practising the skills they needed rather than focusing on exams.

"In the final year you weren't concentrating on exams you were concentrating on how to be a doctor and it was a load of fun and the best year. I was in the hospitals more because I wasn't worried about going home and hitting the books."

### Strengths and weaknesses

There were many different answers to this according to the individuals but the main strengths of the course were seen as engendering graduates with problem solving/research/independent thinking skills, preparation for the role of junior doctor, clinical skills training and the clinical skills resource centre, having various options to explore interests through SSCs.

"the biggest strength for me was the opportunity to do research based things....I do think another strength of the course was - it has given me - and I know it is something they were inspiring to it has given me an independent way of learning.."

"it gives you great skills to be a house officer and from there you need to do it yourself but you have been given the skills to do that.."

"I think the biggest strength is it teaches you to do the job.....you can take a decent history and examination.... you could set up special study modules in all kinds of specialties and we were well supported to follow things that interest you.."

Science teaching was seen as the biggest weakness. Although, many interviewees had individual and often personal suggestions about the programme, there was a general consensus about how the programme could be improved and this was through more "structured" science teaching, particularly in the first year. The most frequently requested extra science tuition was in anatomy followed by pharmacology and pathology. The structure they wanted ranged from more lectures, having physician only PBL facilitators, more exams, more small group sessions with experts, more laboratory teaching and more "formal" teaching in the HARC. Some also suggested there should be a short pharmacy course within the undergraduate programme.

"The weakness is undoubtedly what is not taught, there needs to be more didactic teaching.... the odd extra lecture here and there or more plenary sessions, more organised tutorials and teaching in HARC."

"The weaknesses were lack of guidance and any sort of syllabus or guidelines.. we needed more than one lecture a day in the first year.. anatomy demonstrators and dissection and a syllabus."

Many also commented that they felt undermined by local consultants who were opposed to the reformed curriculum in principle and those from the first cohort in particular cited that it was sometimes difficult to be the first cohort to undertake a brand new programme.

"I am glad I went to Liverpool and I think the university.. is a great place to be a student.. the drawbacks aside the new curriculum was a step in the right direction.. we were the guinea pigs, but we were told that from day one... I am really glad I went through it.."

Nobody called for the PBL to be cut totally from the course, although there were many varying answers about how much of the programme should contain PBL. Those who were least happy with the course wanted more traditional style lectures at the expense of PBL sessions and vice versa. Their happiness with the course correlated with how much "structure" they felt should be included alongside the PBL. Only five said they wished they had studied under a fully traditional programme, though.

Generally, they had enjoyed their time as undergraduates but the majority said the programme could be improved by incorporating the changes they suggested above.

## Discussion

There were some potential limitations to this study. It is possible that the interviews may have attracted people who had either more positive or more negative views of the curriculum. However, a large number were interviewed to account for these possibilities. There were no interviewees in the study who had only positive or negative views about their undergraduate education. Also, it is important to stress, all the graduates were comfortable to say what they felt was good and bad about the course. Although, more interviews could have been arranged, interviewing 45 graduates is more than the (commonly) accepted minimum amount for gaining a consensus on a group of people with similar backgrounds where 10/15 can be seen as enough to gain saturation of themes [19]. A limitation of the study could be that only 45 out of a possible 330 were interviewed. However, the interviews were representative of the gender ratio of the cohorts and covered a wide range of specialties. It is not clear how representative of the cohort as a whole the interviews were, as we do not have career details for all graduates. However, for the questionnaire part of the study [16] which represented 117 doctors and a 35% response rate consisted of 13% surgeons, 37% physicians, 44% GPs, 6% psychiatrists which suggests the sample here may be representative of the cohort as a whole.

The most contentious issue arising from these interviews concerned science knowledge. The majority did feel that as a result of learning sciences in a PBL environment they were lacking compared with graduates from a more traditional lecture based programme and that they had to work harder than traditional graduates taking Royal College exams though this varied from interviewee to interviewee. Many of the postgraduate Royal College exams in the UK are based on traditional exams and the first part of these exams in particular tend to focus in depth on the clinical sciences. However, a recent study has shown that there is no difference in the success rates of Liverpool graduates from the TMC and RMC for part one of the Royal College of Anaesthetists exams [20].

One of the rationales behind curriculum reform was to reduce the "factual burden" on students and this was a deliberate strategy adopted by The University of Liverpool and by the GMC in *Tomorrow's Doctors *[2]. It could be argued that there was a certain contradiction in that many of the interviewees had gathered an understanding of disease processes during their time as an undergraduate yet felt their knowledge was poor. Concern about science knowledge levels in a PBL programme is nothing new [21,22] although often these fears are unfounded as factual knowledge levels between PBL and non PBL graduates have been shown to be similar [23,24]. It is noticeable that physiology was the science which caused the least concern. The graduates may think that a good knowledge of physiology may help with understanding disease processes and physiology may be seen to be suitable for PBL [25]. However, the graduates felt that anatomy and pharmacology, which they believed they had a less of an understanding of may suit a more didactic teaching approach. More research in this area may be needed.

Other medical schools which also use PBL such as McMaster [26] and The University of Sydney [27] have recently introduced more didactic teaching, particularly in anatomy whilst retaining the PBL philosophy of their curricula. Recently, a major review of the curriculum has taken place at Liverpool and it is planned to introduce more "structure" alongside the PBL [28]. Also, after these doctors had graduated some modifications did take place to the programme in 2004 including re structuring of exams and the introduction of a pharmacy course but more reforms are planned. There has been some debate about whether introducing PBL into a medical curriculum can produce graduates who are "lifelong learners" [29] or have self directed skills [30]. These graduates do feel like the Liverpool curriculum has imparted those skills and they feel they are important skills to have. The views of these graduates tie in with other evidence that does show that PBL can encourage self directed continuing learning [31]. However, having more "structure" in the programme such as more lectures, small group sessions or classes in the HARC may take away from these independent, research skills, although many interviewees indicated that the SSCs were useful for gaining research skills. There was a contradiction as many of the doctors said that a main strength of the course was developing these skills but the lack of structured science teaching was the main weakness of the course. Whilst they were happy to have these skills, there was near unanimity that the programme could be improved by having more "structure" in the early part of the course to assist science knowledge acquisition.

The graduates felt that they had been well prepared to work as junior doctors and many aspects of the programme were very useful. In fact in many ways the reformed curriculum was seen as working and meeting its objectives in that the graduates felt they had received excellent preparation to work as junior doctors, utilised the communication skills training, understood the relationship between primary and hospital care, learned history and examination techniques and felt like independent critical thinkers with problem solving skills - all aims of the recommendations in *Tomorrow's Doctors*. This has been corroborated by consultants who supervised them as junior doctors [15]. This was in stark contrast to interviews held with the traditional graduates from Liverpool who said that they felt they were not well prepared to work as junior doctors [13]. One of the reasons the traditional graduates stated for this was they spent the final year learning for exams whereas the RMC graduates were practising the skills for the job and learning the role of junior doctor. Different medical schools vary the amount of "shadowing", but the 8 week "shadow" placement and the final year as a whole at Liverpool seems to have worked very well.

Many were generally happy with the programme. It is unlikely there is a medical curriculum anywhere that the majority of students who study on it are going to be 100% happy. However, there were a small number who very unhappy with the PBL programme so it could be that medical students at the admissions procedure need to be made aware of what type of course they are applying for. It does seem that despite earlier concerns about the amount of General Practice it hasn't had a negative impact on GP recruitment and although some would like the amount of General Practice reduced they did feel there were benefits to GP including understanding the relationship between primary and hospital care. This study adds to the literature than an increase in community teaching can have a positive effect on the attitudes of graduates to general practice [32,33].

## Conclusion

The views of these graduates were very similar to the views these cohorts demonstrated in their first year after graduation [14]. The fact that they could express the same views after more responsibilities as doctors and having made their career choice seems to indicate that undergraduate medical education does have a major and consistent influence on postgraduate experiences. Despite the concern over knowledge base these interviews do show that a reformed curriculum incorporating the recommendation in *Tomorrow's Doctors *can produce graduates who feel they have been very well prepared clinically to train and work as doctors.

## Competing interests

The authors declare that they have no competing interests.

## Authors' contributions

SW conceived and designed the study, managed the evaluation project, analysed and interpreted the data, drafted the article and approved the final version to be published. HOS and DT reviewed and analysed the data, contributed to the first and subsequent drafts and approved the final version to be published.

## Pre-publication history

The pre-publication history for this paper can be accessed here:

http://www.biomedcentral.com/1472-6920/10/65/prepub
